# Evaluation of a semi-automated in vitro feeding system for *Dermacentor reticulatus* and *Ixodes ricinu*s adults

**DOI:** 10.1007/s00436-017-5648-y

**Published:** 2018-01-03

**Authors:** Bettina Böhme, Christoph Krull, Peter-Henning Clausen, Ard M. Nijhof

**Affiliations:** Institute for Parasitology and Tropical Veterinary Medicine, Freie Universität Berlin, Robert-von-Ostertag-Str. 7-13, 14163 Berlin, Germany

**Keywords:** *Ixodes ricinus*, *Dermacentor reticulatus*, Artificial tick feeding

## Abstract

The long feeding duration of ixodid ticks and need for regular blood changes turns the artificial feeding of ticks into a tedious process. To reduce the number of blood changes, a semi-automated system (SAS) for the artificial feeding of hard ticks was developed and evaluated. It consisted of a glass feeding reservoir that can accommodate six tick feeding chambers. A peristaltic pump was used to pump blood through the feeding reservoir, which was changed once daily. Groups of *Dermacentor reticulatus* and *Ixodes ricinus* adults were fed simultaneously in both the SAS and a conventional in vitro feeding system. In the conventional system, feeding chambers were hung inside a glass beaker filled with blood that was replaced twice daily. *Dermacentor reticulatus* adults fed in the SAS obtained significantly higher engorgement weights. Although engorgement rates between both systems were comparable, significantly more SAS-fed females laid fertile egg batches. The egg batch weight of SAS-fed females was also significantly higher. In contrast, the engorgement rate and fecundity of SAS-fed *I. ricinus* were significantly reduced in comparison to ticks fed in the conventional system. This reduction was likely to be caused by fungal infestation, which could spread between feeding chambers in the SAS. Although the SAS reduced the workload compared to the conventional feeding system and showed promising results for the in vitro feeding of *D. reticulatus* adults, measures to prevent fungal infestations in the SAS should be considered in future studies.

## Introduction

Ticks are obligate haematophagous ectoparasites that infest both humans and animals and may act as vectors of pathogens. Two common tick species in Central Europe are *Ixodes ricinus* (Linné, 1758) and *Dermacentor reticulatus* (Fabricius, 1794). *I. ricinus* is the most widespread tick in Central and Western Europe. It has a broad host range and can transmit a wide variety of pathogens, including *Borrelia burgdorferi* sensu lato causing Lyme borreliosis, *Anaplasma phagocytophilum* causing human granulocytic anaplasmosis, *Babesia microti*, *Babesia venatorum* and *Babesia divergens* causing babesiosis in humans and animals and tick-borne encephalitis virus (Jongejan and Uilenberg 2004). *Dermacentor reticulatus* can be found in many parts of Europe, and its distribution in many regions has expanded during the last decades (Rubel et al. 2016). Its veterinary relevance lies in its role as a vector for *Babesia canis* and *Theileria equi*, causing babesiosis in dogs and horses, respectively, but it has also been associated with the transmission of human pathogens such as *Rickettsia slovaca* and *Rickettsia raoultii*, causing tick-borne lymphadenopathy (TIBOLA), and a flavivirus causing Omsk haemorrhagic fever (Jongejan and Uilenberg 2004).

For research on ticks and tick-borne pathogens, the use of live ticks is often required, and tick colonies are routinely maintained in laboratories worldwide (Levin and Schumacher 2016). All life stages are usually fed on experimental animals, which most closely resemble the natural situation and is highly efficient, but is also associated with ethical concerns. An alternative to the feeding of ticks on animals is the artificial feeding of ticks, which provides interesting potential research applications and also adheres to the 3R principles to ‘Reduce, Replace and Refine’ the use of experimental animals in research (Russel and Burch 1959). However, artificial tick feeding is associated with drawbacks that have restricted their application, as not all tick life stages and species, in particular those with shorter mouthparts, can be fed successfully in vitro, and a significant reduction in tick fertility has been reported for artificially fed ticks. Finally, artificial tick feeding requires a blood donor, expertise and considerable manual labour (Kuhnert 1996; Krull et al. 2017).

Artificial tick feeding usually requires a synthetic membrane or animal skin, through which the ticks feed on a provided blood source. The blood used for in vitro feeding needs to be constantly heated to the host body temperature to accommodate tick feeding (Voigt et al. 1993), which at the same time provides optimal conditions for bacterial and fungal growth and decay of the blood meal when blood collection and/or in vitro tick feeding are performed under unsterile conditions. To prevent this from happening, the addition of broad-spectrum antibiotics and fungicides to the blood and a regular change of the blood meal, typically twice daily but up to six times per day, are commonly employed for in vitro tick feeding (Waladde et al. 1993; Kuhnert et al. 1995; Krober and Guerin 2007). As tick feeding may take up to several weeks (Jones et al. 1988), the manual labour associated with artificial tick feeding is considerable.

An attempt to partially automate the feeding of haematophagous arthropods to reduce the associated workload of manual blood changes has been described (Kuhnert et al. 1998). Although one of the experiments performed using this system resulted in engorgement weights and fertility rates that were not significantly different from *Amblyomma hebraeum* adults fed in vivo on cattle, the number of ticks that could be fed was limited and the approach was not pursued further. Another interesting artificial feeding approach was published for colony maintenance of the human head louse, *Pediculus capitis*. In this system, blood and saline flowed alternately through a heated feeding reservoir on which up to 13 small feeders with a diameter of 1.25 cm could be placed (Takano-Lee et al. 2003).

In this study, the use of a novel semi-automated artificial tick feeding system (SAS) was evaluated for feeding *D. reticulatus* and *I. ricinus* adults. The SAS uses the same glass feeding units as previously used for a study on the optimization of in vitro feeding for *D. reticulatus* (Krull et al. 2017). By performing the tick feedings simultaneously in both SAS and this conventional system, we were able to compare the feeding success and fertility of the ticks between both systems.

## Material and methods

### Ticks

Adult *I. ricinus* and *D. reticulatus* ticks were collected by flagging the vegetation of fallow land and forests in and near Berlin. Ticks were stored in glass containers with pierced lids in a desiccator containing saturated MgSO_4_ solution, providing a relative humidity of approximately 90% at room temperature and a 15:9-h light/dark cycle.

To evaluate the efficacy of the SAS in comparison to the conventional system, adult ticks were randomly placed in two groups for subsequent artificial feeding. Artificial feeding experiments in the SAS and the conventional system were performed simultaneously for each species. The *D. reticulatus* experiments were performed in 2014 and the *I. ricinus* experiments in 2016. The bovine blood used in both systems within a single experiment originated from the same animal. For *D. reticulatus*, seven female ticks were used per feeding chamber and two experiments with six feeding chambers for each system were performed, resulting in a total number of 84 females for each tested system. For *I. ricinus*, ten females were used per feeding unit in the first experiment, and nine females per feeding unit were used in the second experiment, which resulted in a total number of 114 females per system tested.

### Conventional artificial tick feeding system

Preparation of blood, tick feeding units and the blood change procedure was performed as previously described (Krull et al. 2017). Feeding units with *I. ricinus* were kept in an incubator (ICH256C, Memmert, Düsseldorf, Germany) at 20 °C, 80% RH, 2.5% CO_2_ and a 15:9-h light/dark cycle. For logistical reasons, the feeding of *D. reticulatus* was performed in a waterbath set at 38 °C and a 15:9-h light/dark cycle. Additional CO_2_ was not provided for the *D. reticulatus* feeding experiments.

### Semi-automated feeding system

**Fig. 1:**
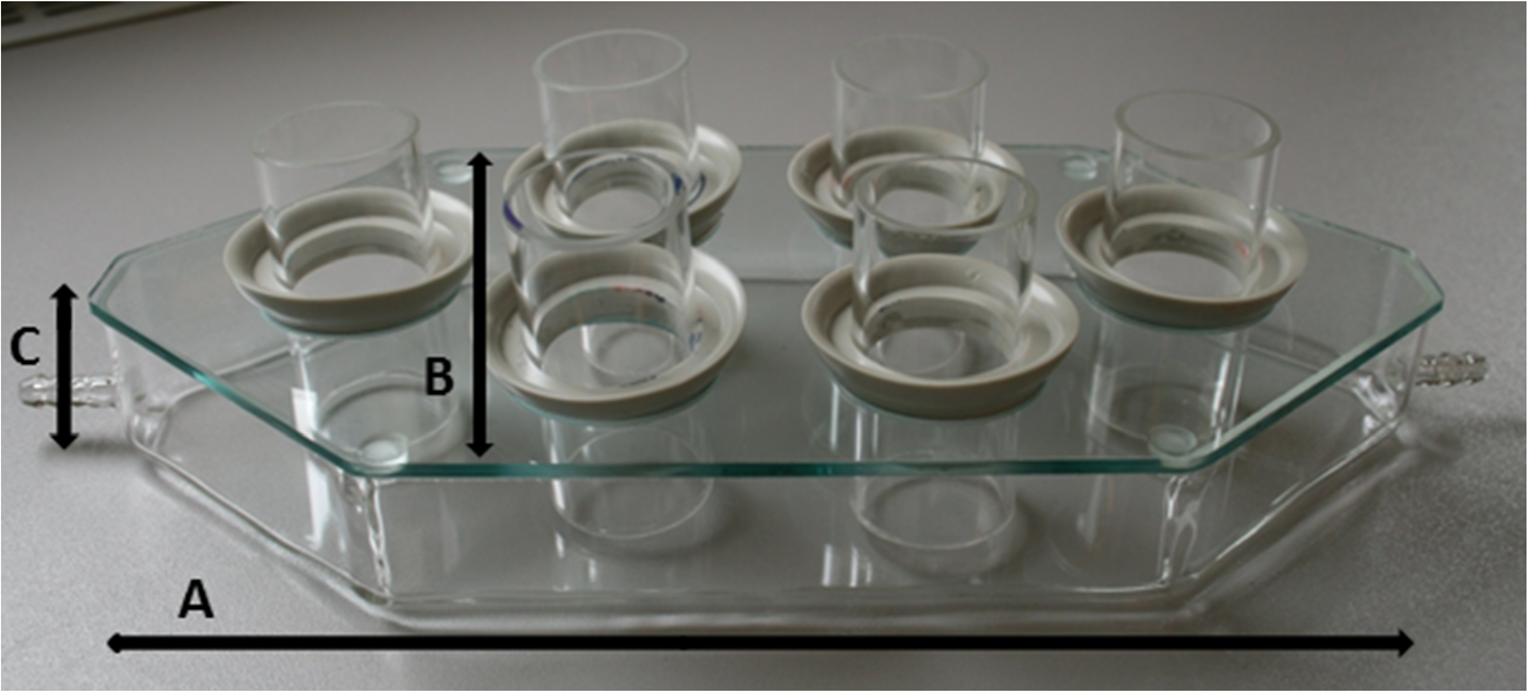
Feeding reservoir with inserted tick feeding units. Membranes, tubing and lids are not shown. A = 270 mm, B = 125 mm, C = 30 mm

**Fig. 2:**
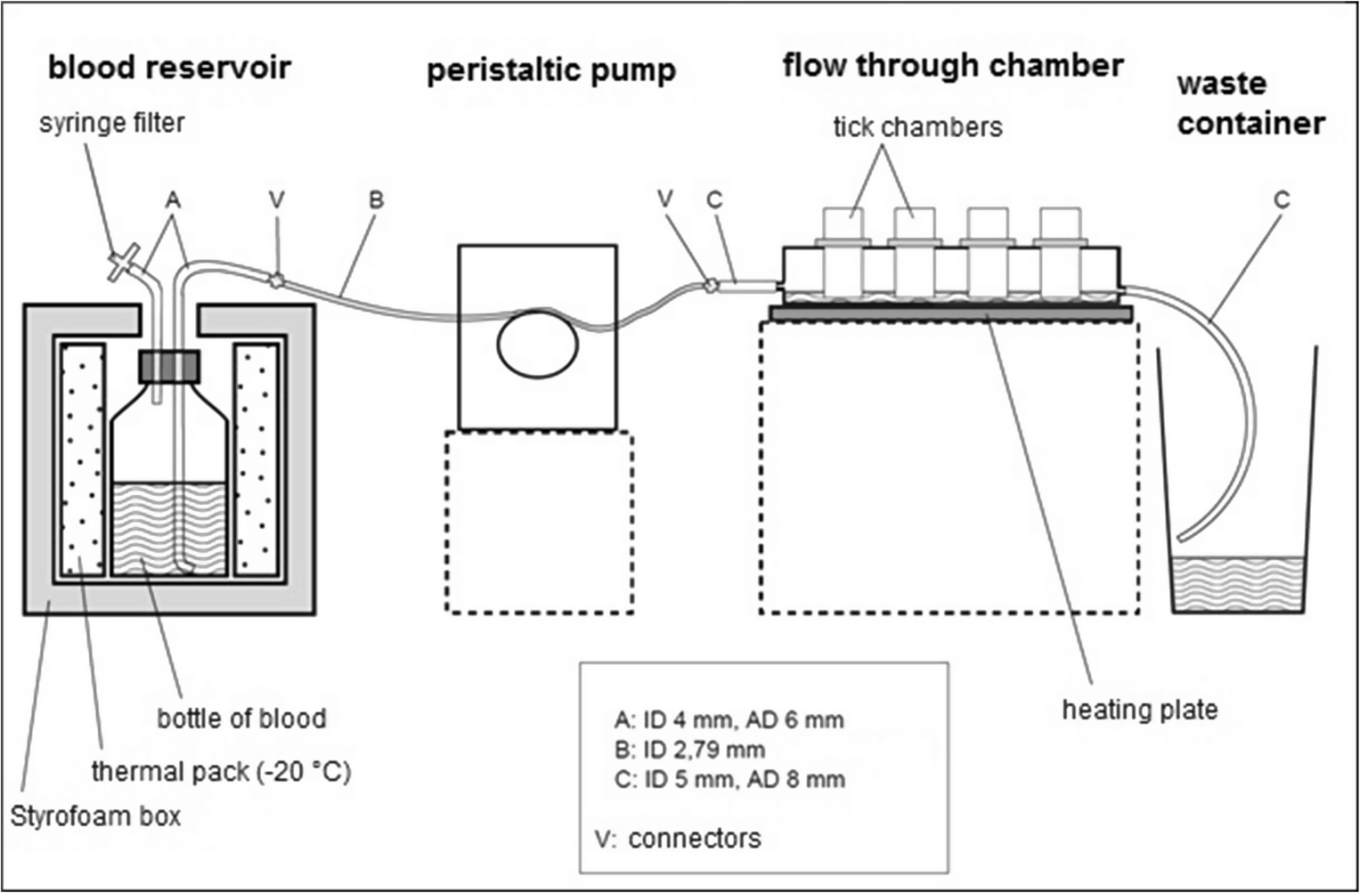
Schematic drawing of the semi-automated artificial tick feeding system. ID inner diameter, AD outer diameter of tubing

The SAS consisted of a custom-made hexagonal borosilicate glass feeding reservoir (Technische Universität Berlin, Germany) which featured one outlet of 5 mm diameter on both ends (Fig. 1). The feeding reservoir was covered by a separate hexagonal glass plate containing six holes of 40 mm diameter, with a minimum distance of 20 mm between the holes. A single glass feeding unit, identical to the one used in the conventional system, could be hung in each hole using a rubber ring with an inner diameter of 32 mm (Lux, Wermelskirchen, Germany) (Fig. 2).

The blood reservoir consisted of a 1-L bottle which contained 600 mL blood supplemented with heparin (20 IU/mL, Ratiopharm, Ulm, Germany), glucose (2 g/L blood, Carl Roth, Karlsruhe, Germany), adenosine triphosphate (ATP) (51 mg/mL, Carl Roth) and gentamycin (Carl Roth) (5 μg/mL). Gentamycin and ATP were added to the blood reservoir just before use. The blood reservoir was placed inside a Styrofoam box and refrigerated with frozen ice packs that were changed daily. Blood was pumped from the bottle using a peristaltic pump (Reglo ICC, Ismatec, Wertheim, Germany) through silicone tubing into the feeding reservoir at a rate of 0.2 mL/min. A second tube with a 0.2-μm syringe filter (Carl Roth) was connected to the bottle cap for pressure equalisation. The outlet of blood from the feeding reservoir occurred passively through a silicon tube which led to a receptacle containing the waste blood that was discarded. The blood meal was heated to 37 °C by placing the feeding reservoir on top of a heating plate (Hot Plate 062, Labotect, Göttingen, Germany). *Dermacentor reticulatus* feeding was performed in the laboratory at a room temperature of 20 °C and a 15:9-h light/dark cycle without additional CO_2_. Damp cotton balls on top of the tick chambers provided the ticks with a RH of approx. 70%. All feeding experiments for *I. ricinus* were performed in an incubator (ICH256C, Memmert) at 20 °C, 80% RH, 2.5% CO_2_ and a 15:9-h light/dark cycle. The slow influx of refrigerated blood into the heated flow-through chamber ensured a constant temperature. Tick chambers were hung into the holes of the glass plate covering the feeding reservoir so that the underside of the feeding chambers was immersed in blood. The environmental conditions inside the feeding chambers for the second *D. reticulatus* experiment and both *I. ricinus* experiments were recorded by an iButton (Hygrochron, Maxim Integrated, San Jose, USA) that measured temperature and RH at 30-min intervals.

Blood change for the SAS was done once per day. The feeding reservoir was disconnected and placed on a heating plate (Labotect) in the laboratory. A new sterile feeding reservoir was connected to the pump and filled with fresh blood at a rate of 30 mL/min. The blood was left to be warmed whilst the feeding units were examined for detached ticks and membrane integrity and rinsed with pre-heated sterile 0.9% NaCl solution. The rinsed feeding units were placed in a new sterile feeding reservoir lid which was placed on top of the feeding reservoir. Every second day, the blood reservoir was depleted and replaced with a new bottle and new tubing. The blood reservoir bottles, silicon tubing, feeding reservoirs and glass plates covering the feeding reservoir were washed and autoclaved before use.

### Statistical analysis

Statistical analyses were performed using SPSS software. Engorgement rates and rates of females producing fertile eggs were evaluated using Fisher’s exact test. Engorgement masses and egg batch masses were evaluated using *t* test or Mann-Whitney *U* test, depending on normal distribution of the data.

## Results

### *Dermacentor reticulatus*

In the conventional system, 26.2% females engorged (22/84), which was not significantly different from the number of ticks which engorged in the SAS (27.4%, 23/84, *p* = 1.00, Fisher’s exact test). The average mass of engorged females differed significantly between both systems, with 185.2 ± 78.2 mg in the conventional group and 269.6 ± 105.1 mg in the SAS group (*p* = 0.004, *t* test). The number of engorged females producing fertile egg batches differed significantly: 22.7% (5/22) in the conventional group and 87.0% (20/23) in the SAS group (*p* < 0.0001, Fisher’s exact test). The mass of the egg batches was also significantly different between both groups (80.0 ± 37.8 mg vs. 123.0 ± 71.4 mg, *p* = 0.031, *t* test). The average feeding duration, defined here as the time between placement of the tick in the feeding chamber and its detachment as an engorged specimen from the membrane, was 10 ± 3 days in the conventional group and 10 ± 2 days in the SAS group (Table 1). iButton data from the second experiment showed small differences in the microclimate inside the tick feeding unit. The RH and temperature were 78.0 ± 2.4% and 38.1 ± 0.2 °C in the conventional system and 74.2 ± 3.0% and 36.4 ± 0.4 °C in the SAS.

**Table 1:** Effects of in vitro feeding using the conventional and semi-automated feeding system (SAS) on the engorgement and fecundity of *Dermacentor reticulatus* and *Ixodes ricinus* females

Species	Experimental group	Engorgement rate	Engorgement mass ± SD (mg)	Females producing fertile eggs	Egg batch mass ± SD (mg)	Time to repletion (day)
*D. reticulatus*	Conventional	26.2% (22/84)	185.2 ± 78.2^b^	22.7% (5/22)^c^	80.0 ± 37.8^e^	10.1 ± 2.5
	SAS	27.4% (23/84)	269.6 ± 105.1^b^	87.0% (20/23)^c^	123.0 ± 71.4^e^	9.8 ± 2.1
*I. ricinus*	Conventional	80.7% (92/114)^a^	217.0 ± 95.6	66.3% (61/92)^d^	56.5 ± 33.4	10.8 ± 2.2
	SAS	20.2% (23/114)^a^	204.5 ± 131.6	30.4% (7/23)^d^	53.9 ± 30.4	11.4 ± 1.6

^a^Significant difference (Fisher’s exact test, *p* < 0.0001)

^b^Significant difference (*t* test, *p* = 0.004)

^c^Significant difference (Fisher’s exact test, *p* < 0.0001)

^d^Significant difference (Fisher’s exact test, *p* = 0.0037)

^e^Significant difference (*t* test, *p* = 0.031)

### *Ixodes ricinus*

iButton data showed that the RH inside the tick feeding units averaged to 61 ± 9% in the conventional system and to 54 ± 10% in the SAS, and thus remained lower than the set incubator RH of 80%. Of the ticks fed in the conventional system, 80.7% engorged (92/114). This was significantly more than the number of engorged ticks in the SAS system, where 20.2% (23/114, *p* < 0.0001, Fisher’s exact test) engorged. The average mass of engorged females was not significantly different, with 217.0 ± 95.6 mg in the conventional system and 204.5 ± 131.6 mg in the SAS (*p* = 0.218, Mann-Whitney *U* test). The number of engorged females producing fertile egg batches differed significantly: 66.3% (61/92) in the conventional system and 30.4% (7/23) in the SAS (*p* = 0.0037, Fisher’s exact test). No significant difference could be found between the deposited egg mass of 56.5 ± 33.4 mg by females fed in the conventional system and 53.9 ± 30.4 mg by females fed in the SAS (*p* = 0.984, Mann-Whitney *U* test). The average feeding duration was 11 ± 2 days in the conventional group and 11 ± 2 days in the SAS group. A noticeable difference was fungal infection, which affected five of six feeding units in the SAS in the first experiment and four of six feeding units in the second experiment, whereas fungal infections were not observed in the conventional system. Of the seven SAS-fed ticks that laid fertile egg batches, six were fed in feeding units that were not affected by fungi.

## Discussion

We previously demonstrated that the in vitro feeding of *D. reticulatus* adults could significantly be improved by exposing ticks to increased CO_2_ levels during feeding and by adding extra glucose to the blood meal (Krull et al. 2017). In the study presented here, we focused on the evaluation of a system that would significantly reduce the labour associated with in vitro tick feeding, whilst maintaining desirable feeding and reproduction results. This SAS was considered to be semi-automated, since a change of the feeding reservoir once per day was necessary to prevent the accumulation of blood coagulations inside the reservoir. Nonetheless, the workload could be reduced by half compared to the conventional manual feeding system in which the blood-filled beakers were changed twice daily. A drawback of the SAS is the larger volume of blood required due to the steady flow of blood through the system. In the setup presented here, a reservoir of 600 mL provided six feeding chambers in the SAS with blood for a period of 48 h. In the conventional system and within the same period, six feeding chambers required 120 mL blood, i.e., five times less than the SAS. The use of the SAS for the artificial feeding of ticks would therefore be of most interest in situations where the blood supply is not a limiting factor. We refrained from the re-use of blood through a conversion of the SAS into a circulatory system, as this was considered likely to result in obstructions of the tubing caused by blood coagula and facilitate the further growth of any contaminants present.

The SAS was most successful for the feeding of *D. reticulatus* adults, as these obtained a significantly higher engorgement weight and laid more fertile eggs than their counterparts fed on the same blood source in the conventional system. Ticks gain most of their weight during the rapid phase of engorgement, which usually lasts 1–2 days on a natural host and is associated with enhanced salivation and increased blood flow to the tick bite site (Tatchell et al. 1972; Fielden et al. 1999). Secreted tick saliva is more likely to disperse in the SAS due to the steady stream of blood below the membrane compared to the conventional system. This enhanced dispersal might attribute to the increased weight gain of *D. reticulatus* ticks observed in the SAS, as it more closely mimics the natural situation. Increased concentrations of salivary components around the mouthparts of ticks feeding in the more stagnant conventional system might have inhibited further feeding (Kemp et al. 1982) and the uptake of nutritional blood constituents, leading to a suboptimal weight gain and lower engorgement weights. Although small differences in RH and temperature inside the tick feeding units between both systems were observed, we consider it unlikely that these differences explain the differences in engorgement weight and fecundity. The artificial feeding of *Rhipicephalus sanguineus* at 27 and 37 °C, a far greater temperature difference than we observed, was recently shown to have no significant effect on the mean weight gain of ticks (Valim et al. 2017).

The engorgement rates of *D. reticulatus* females fed in this study were relatively low for both the conventional and SAS method (26.2–27.4%), whereas the engorgement rate of *I. ricinus* females fed using the conventional in vitro system (80.7%) was much higher. The precise reasons for this are unknown, but features of the in vitro system such as membrane structure and attachment stimuli used may have played a role. It should also be noted that *I. ricinus* is a prostriate tick; all females had ample access to males prior to their introduction in the tick feeding chambers and it is likely that copulation had already taken place, obviating mating during feeding (Kiszewski et al. 2001) which might have facilitated the in vitro engorgement for this species.

Although we did not observe significant differences of the feeding duration for *I. ricinus* between the conventional in vitro fed group and the SAS fed group, the duration was longer than the 6–8 days, which *I. ricinus* typically needs to engorge on cattle (data not shown). Other groups have also reported a longer duration of tick feeding in vitro compared to ticks fed on animals (Kuhnert et al. 1995; Liu et al. 2014). We observed that some ticks placed in the tick feeding chamber did not attach immediately, but needed several days until attachment and feeding took place. This may in part explain the longer in vitro feeding duration observed here. Encouragement of questing behaviour and swift tick attachment through the provision of additional stimuli such as CO_2_, host or synthetic odours, visual cues, vibrations and membranes that more closely mimic the natural hosts’ skin could shorten the feeding duration and further improve the artificial feeding of ticks (Waladde et al. 1996; Krull et al. 2017; Trentelman et al. 2017).

The most plausible explanation for the reduced feeding success and fecundity of *I. ricinus* in the SAS were the severe fungal infestations of the membranes, which affected 9 of 12 feeding chambers. It is likely that spread of the fungal contamination between the different feeding chambers was facilitated by the flow of blood within the SAS. The origin of the contamination remained unclear, but blood or ticks were possible sources. Bovine blood was collected during exsanguination at a slaughterhouse and contact with the natural mycoflora of cattle hair and skin could not be excluded.

Ticks, including *I. ricinus* and *D. reticulatus*, have been reported to harbour various species of fungi (Samsinakova et al. 1974; Bell 1980; Kalsbeek et al. 1995). Kuhnert observed that fungal growth on the artificial feeding membranes started predominantly on cement cones of tick feeding sites and identified these fungi as *Aspergillus fumigatus* and *A. ochraeus* (Kuhnert 1995). Although measures were taken to minimise the risk of contamination during the in vitro feeding process in the laboratory, an environmental contamination cannot be fully excluded either. Surface sterilisation of ticks prior to artificial feeding, or the addition of fungicides to the blood meal may solve this problem. Since treatment of ticks with antibiotics may have a negative impact on tick fitness and fecundity (Zhong et al. 2007; Narasimhan et al. 2014) and potentially symbiotic fungi have been reported from *Ixodes* ticks (Benoit et al. 2005), the addition of biocidal products to the blood meal may have negative consequences for the ticks, which should be carefully assessed.

In conclusion, the SAS evaluated in this study showed promising results for the feeding of *D. reticulatus* adults. *Dermacentor reticulatus* ticks fed using the SAS obtained significantly higher engorgement weights and reproduction rates compared to ticks fed in vitro using the conventional system. In addition, the SAS required less manual work. By contrast, the poor feeding results for *I. ricinus* and increased fungal contamination in the SAS found when feeding this tick species also revealed a weakness of the SAS. The prevention of fungal contamination in the SAS should have priority in further studies to examine its suitability for feeding additional tick species.
